# The Importance of Long-Term Social Research in Enabling Participation and Developing Engagement Strategies for New Dengue Control Technologies

**DOI:** 10.1371/journal.pntd.0001785

**Published:** 2012-08-28

**Authors:** Darlene McNaughton

**Affiliations:** Discipline of Public Health, Flinders University, Adelaide, South Australia, Australia; International Vaccine Institute, Korea

## Abstract

**Background:**

In recent years, new strategies aimed at reducing the capacity of mosquito vectors to transmit dengue fever have emerged. As with earlier control methods, they will have to be employed in a diverse range of communities across the globe and into the main settings for disease transmission, the homes, businesses and public buildings of residents in dengue-affected areas. However, these strategies are notably different from previous methods and draw on technologies that are not without controversy. Public engagement and authorization are critical to the future success of these programs.

**Methodology/Principal Findings:**

This paper reports on an Australian case study where long-term social research was used to enable participation and the design of an engagement strategy tailored specifically to the sociopolitical setting of a potential trial release site of *Wolbachia*-infected *Aedes aegytpi* mosquitoes. Central themes of the social research, methods used and conclusions drawn are briefly described. Results indicate that different communities are likely to have divergent expectations, concerns and cultural sensibilities with regard to participation, engagement and authorization.

**Conclusions/Significance:**

The findings show that a range of issues need to be understood and taken into account to enable sensitive, ethical and effective engagement when seeking public support for new dengue control methods.

## Author Summary

A number of new strategies are emerging to combat dengue fever. Many are notably different from earlier methods and not without controversy. Public participation, engagement, enablement and authorization are recognized as critical to the success of field trials and future use and acceptability of new strategies. It is well established in cultural anthropology and public health that interventions often fail because of a lack of understanding of the local context, lay knowledge of the disease, and community, local or regional expectations around engagement. In this paper we demonstrate the critical importance of using long-term social research to address these issues and to design engagement strategies and communication materials that are tailored specifically to the needs, expectations and concerns of residents at a potential release site. We report on the results of an extensive and systematic social research and engagement program undertaken in the two years prior to the first successful release of *Wolbachia*-infected *Aedes aegypti* mosquitoes in Cairns, northern Australia.

## Introduction

In the last 30 years there has been a revival of interest in community or public engagement and participation; in including lay people in the development and decision making surrounding scientific research, regulatory procedures, policies and programs [1]. This renewed interest is evident in many democratic, industrialized nations and reflects “a more general concern with developing a non state-based sphere of ‘the political’ and with nurturing local autonomy” [2]. For many of its supporters this is a welcome move away from earlier top-down, government and expert-centered approaches. Alongside these developments, the concept of the environment has also begun to feature centrally in public consciousness, in political action and at the level of policy and there has been a concomitant increase in the scrutiny of biological control programs, biotechnologies and genetic applications [2], [3], [4], [5], [6]. This in turn has led to a heightened awareness of the potential conflicts and public concerns that might accompany their use.

It is within the context of these increasingly globalized political developments that several new strategies aimed at reducing the capacity of mosquito vectors to transmit dengue fever have emerged. Some focus on genetic modification; others, like the program reported here, on biological control. However, taking science (and scientists) out of their laboratories and into the environment has consequences for local residents, whose homes and places of work, leisure, education and worship become outdoor laboratories for testing the efficacy of these methods or an open release site for a strategy. The ethical complexities of these field trials and environmental releases are considerable, and regulatory approval, collaborative partnerships, public engagement and authorization are seen by many as essential ethical requirements that should be secured *before* undertaking open field releases [6], [7], [8], [9], [10], [11], [12], [13].

Public engagement is now almost obligatory in the bioscience and biotechnology fields. However, there is great variation in how this is understood, and in the expectations and motivations that drive programs [14]. For example, some programs reflect the view that those most affected by decisions have the right to participate in them, others emphasize the need to reduce conflict (actual or expected), or see engagement as a way to raise new questions about these strategies that will improve implementation and acceptability [14]. The approach described in this paper encompasses all these motives. However, it also aimed to *enable* the public to take a significant role in shaping the program, including the scientific research, determining how engagement should proceed, what authorization might look like and, fundamentally, how dengue fever should be managed in their community. This was achieved through formal discussions with as wide a range of people as possible across a large and diverse population. The emphasis on collaboration, dialogue, information sharing and shared decision making brings this approach close to what is commonly known in the bioscience field as Public Technology Assessment (PTA), and in public health as public engagement [14], [15].

Cultural anthropology has had a long involvement in public health. As a disciplinary framework, it focuses on the broader historical and sociopolitical context and on local knowledge, cultural sensitivity and grounding in community [16]. It is fundamentally a bottom-up approach that “allows the use of indigenous knowledge and community resources, and also makes local persons and community groups co-responsible for developing policies, programs, and activities for improving health” [16]. As a theorizing activity it brings several critical insights that are relevant here. The first is that there is no single “one size fits all” approach to public participation or engagement that can be applied universally; in other words, what works in one place may not work in another. Perhaps the most compelling reason for this is that different communities have divergent expectations, concerns, political proclivities, structures and cultural sensibilities that need to be understood, respected and taken into account if one is to engage sensitively, ethically and effectively.

The second, related, insight is that all human knowledge is culturally and historically shaped, including people's understandings of disease, illness and preventive measures [17], [18], [19]. This has led many to suggest that health interventions have been failing, in part because they are based on a limited awareness of the complexity of the local cultural context and of the complexity of public interpretations and understandings of disease, health interventions and education [19]. This insight has been taken up by a number of scholars with regard to public health programs and initiatives for dengue and malaria [20], [21], [22], [23].

It is widely accepted that “dengue is a complex disease and attempts to control and prevent it do not take into account the fact that we still do not know what dengue is culturally and what it means for individuals in their everyday lives” [24], or between regions [25], [26]. Furthermore, financial and time constraints have led to an over-reliance on Rapid Assessment techniques and Participatory Action Research procedures in this field [27]. The real cost of this “is the loss of contextual information, and the probable oversimplification of behavior due to the brevity of fieldwork and the lack of participant observation which enhances, and indeed is the one means to ensure, validity of data” [27]. In other words, while quick and cheap (and sometimes the only options available because of budgetary and time constraints) these techniques produce a limited and less reliable understanding of the sociopolitical context and of lay understandings of disease, both of which are critical to improving the success of interventions and engagement [18], [19], [24], [25].

In this paper we report on the use of long-term, systematic social research to enable participation and the design of an engagement strategy tailored specifically to the particular cultural, sociopolitical setting of a given region. Based on the open field release trials of *Wolbachia*-infected *Aedes aegytpi* into suburban areas of Cairns, we briefly describe the methods used to determine the nature of the sociopolitical context, lay knowledge of dengue fever, concerns about the initiative, and expectations about engagement and authorization. We then detail some of the key findings, highlighting the particular requirements, assumptions and expectations of the multiple publics that were later used to design an engagement strategy and establish a reference group, and which led to new experiments being undertaken and an independent assessment made of the science.

It will be argued that long-term social research provided important insights into the nature and complexity of the community, which in turn played a critical role in the success of the initiative. It allowed staff to enable participation and work closely with a large number of local residents to identify their questions, concerns and expectations and determine collaboratively how these might be responded to, well in advance of a release. The results of this research were then used to develop a unique large and inclusive engagement strategy, and communication materials that were targeted, culturally appropriate and comprehensible to those being asked to decide how they wanted to manage dengue fever and assess a new method for control. Because the engagement strategy was based on participation and on evidence collected systematically over a 12-month period (2008–2009) it is likely to be more reliable than shorter investigations as a mechanism for enabling public involvement and communicating a complex medical and scientific story. Significantly, formal public engagement was undertaken the following year (June 2009–July 2010). A reference group was established, community involvement and support developed and strengthened, with open release field trials commencing in January 2011.

## Methods

### Setting

The *Wolbachia* strategy aims to “manipulate mosquito populations to make them incapable of transmitting dengue viruses between people” (www.eliminatedengue.com). Researchers had previously demonstrated that the naturally occurring insect bacterium *Wolbachia pipientis* could be transferred from the fruit fly *Drosophila melanogaster* into the *Ae. aegypti* mosquito [28], [29], [30]. They established that the bacterium not only shortened the lifespan of the mosquito but also had a blocking effect on the replication of some dengue viruses [31], [32]. These properties would, in all likelihood, greatly reduce the mosquito's capacity to transmit the virus. Following these and other developments, it was clear that open field-testing experiments would be required in the future. The case study described below focused on the Cairns region (16°55′32″S, 145°46′31″E) with special emphasis on two potential release sites, the southern suburb of Gordonvale (population 4,420) and the northern suburb of Yorkeys Knob (population 2,684) (see Table S1) [33].

### Qualitative and quantitative methods

It is generally accepted that qualitative research methods are the most appropriate mechanism for gauging the views of a population about implementation. In part, this is because of the great importance and emphasis given to context and the creation of empirical evidence through the documentation of knowledge and attitudes in their particular geopolitical setting. The social research that informed the development of the engagement phase is based on an anthropological or ethnographic research design and was undertaken over an 18-month period (2008–2009). It is widely accepted that this focus on the local, ethnographic context and the systematic documentation of evidence over an extended period of time greatly increased the validity of the anthropological research. The study used qualitative and quantitative data collection strategies, including ethnographic (informal) interviews, participant observation, semi-structured in-depth interviews, historical research, focus groups and quantitative telephone surveys. An overview of the research activities undertaken at each stage, the number of participants and recruitment strategies is provided here (see Table S2). The study received ethics approval from the James Cook University Ethics Committee, reference H2250; written consent was obtained for interviews and focus groups, and verbal consent for the telephone surveys, which was documented on the interview guide.

The community profile, historical research, analysis of health authority data, focus groups with mosquito control staff (n = 2), ethnographic (n = 40) and in-depth interviews (n = 10) were completed first. The results were used to inform a series of focus groups (n = 9) with community members on key issues outlined in Table S2, and later into the development of two telephone questionnaires, administered in 2009 and 2010. The stakeholder list was developed alongside these phases. Purposive recruitment (by invitation) using predetermined criteria relevant to the research aims was used for the focus groups with mosquito control staff and in-depth interviews with local leaders. These included politicians, Indigenous elders, mosquito and dengue experts and prominent community leaders from Yorkeys Knob and Gordonvale. Newspaper advertisements were used to recruit members for the community focus groups while respondents for the telephone surveys were recruited by calling randomly generated telephone numbers and further selecting (by age and gender) to ensure a representative sample. Ethnographic interviews were informal, occurring spontaneously and often conducted in the homes or backyards of local residents. In-depth interviews and focus groups were semi-structured and more formal, because key themes had been identified in the ethnographic interviews and historical research. The qualitative data were coded and analyzed with the qualitative software program NVivo8, using open and focused coding. Briefly, this involves describing the full range of themes arising from the data and then moving to an interpretation of the data from within the knowledge that is already present.

Recurring themes from the qualitative research were explored further using quantitative measures (telephone surveys) and representative samples that produced results with a 95% confidence level of ±5.6% [34], [35]. As such, the findings presented here should not be seen as isolated research activities, but as a body of interconnected data developed over time using iterative processes and then contextualized, triangulated and cross-checked using larger, more representative samples in the form of telephone surveys.

One of the first steps was to examine the complexity of the local sociopolitical context, together with public interpretations and understandings of the disease, the risk it was thought to pose and what the public wanted to do about it. A comprehensive stakeholder contact list and a community profile of the region, its history (Indigenous and non-Indigenous), settlement, development and industries were created. The profile included a history of dengue management and biological control, and a sociodemographic profile of the population's education, income, age, gender, occupation, housing, religion and ethnicity.

The key issues explored in the research were: (1) the nature of the sociopolitical context (local, regional, national); (2) lay knowledge and history of dengue fever; (3) lay knowledge and history of biological control; (4) engagement; (5) authorization; and (6) acceptability and non-acceptability of the new method and the release (Table S2). A *selection* of the key findings used to enable participation and develop an engagement strategy and related communication and education materials is provided below.

## Results

### Understanding the sociopolitical context

It was evident from studies undertaken by the local health authority that dengue was a major issue of concern for many residents. The sociodemographic and historical research allowed identification of groups who had been marginalized historically and those who were currently influential, politically and/or economically. It also provided important insights into the diversity and mobility of the local population that helped to ensure greater equity in engagement. For example, the region experiences high levels of mobility. Australian Census data (2006) suggested that 48% of respondents were *not* living at their current address 5 years earlier, and a significant number of these had moved from regions without dengue [33]. Several insights into the potential release sites also became apparent. Gordonvale, for example, had a significantly larger proportion of Indigenous people and residents aged over 65 years than the national average, while Yorkeys Knob had a younger population and a larger proportion of one-parent families and families without children (see Table S3) [33]. In both suburbs, most residents were born in Australia but many were second or third generation migrants.

The informal ethnographic and in-depth interviews with residents and local leaders revealed that Gordonvale residents had a strong sense of local identity, and the community structure was more similar to a small township than a suburb of Cairns. A number of well-respected and active community groups and leaders were identified, including individuals who did not hold any official roles but were highly regarded and trusted. There were several active civic, commercial and church-based organizations, a local Chamber of Commerce, a farmers' association and a dynamic land management/environment group. In contrast, Yorkeys Knob functioned less like a township and more like a residential suburb of Cairns, with fewer civic, commercial, church-based or community groups. However, the informal interviews revealed that it did have an active and well-respected Residents' Association with a history of resisting commercial developments thought to reduce the natural beauty and family-friendly atmosphere of the suburb.

### Local knowledge of dengue, the risk it poses and how to control it

To develop a strong foundation of knowledge about the history and management of dengue fever, past media campaigns were reviewed and the current roles and responsibilities of those involved in dengue management, education, health promotion and research identified. Any underlying tensions or conflicts between organizations or individuals were also identified. This research built awareness of the nature, history and politics informing local dengue education and management and enabled a more informed, constructive and effective collaboration and engagement with these organizations.

Research into lay knowledge of dengue fever, its transmission and health implications produced many significant insights, especially with regard to perceptions of risk and the existence of local ethno-entomologies [36], [37]. For example, in the 2009 telephone survey (n = 300) 78% of residents were “concerned” or “very concerned” about dengue fever and 78% thought its presence in the region had increased. Participants across the nine focus groups (n = 89) and in-depth interviews (n = 40) also had moderate to high levels of concern about the threat of dengue and the need to control the disease. However, they knew little about the extent and nature of the threat and its impact in neighboring countries.

When information about the scale of the problem and the increasing ineffectiveness of current controls was provided at the end of each focus group or interview, we observed that the level of individual concern appeared to increase, as did comments about the need to do more to control the disease. Clearly, residents needed more information about dengue, its risks, the global increase in disease incidence and the challenges of dengue management before they could fully assess whether a trial of the *Wolbachia* strategy was acceptable to them. Improving awareness of these became a central theme in future engagement, communication and education.

It was noted across all the qualitative data that while residents invariably associated the “dengue mosquito” (as it is known locally) with transmission of the disease, other details of the transmission process, virus development and symptoms were not well understood. Many people believed that the dengue virus occurred naturally in the insect and that it was inherited. Residents consistently described encountering “dengue mosquitoes” – “the ones with the black and white stripes” – in a wide variety of habitats and actively challenged health messages stating that suburban backyards were its primary habitat. Most people assumed that all mosquitoes fly considerable distances and spoke of “dengue mosquitoes” as ubiquitous in the landscape, asserting that they live and breed in and around the home *and* in bodies of water such as swamps, creeks and puddles [37].

The key message in regional health campaigns has focused extensively on the appellation “dengue mosquito” and the descriptor “the black and white striped mosquito” but this appears to have created or inadvertently reinforced the idea that the virus occurs naturally in the mosquito. Furthermore, the descriptor “black and white stripes” refers to visible features common to many local species found in a variety of habitats. Its use may have developed or reinforced the view that dengue-infected mosquitoes are ubiquitous in the landscape [37]. Re-analysis of qualitative survey data collected by the local health authority stretching back to 2004 indicated that ideas about the ubiquity of *Ae. aegypti* and its presence in a range of habitats has been present in the population for some years [37].

As these few examples indicate, the results outlined the scale and nature of lay entomologies and etiologies of dengue. Collectively, they strongly suggested that many residents would assume that *Wolbachia*-infected *Ae. aegypti* would also inhabit a range of locations. In fact, this widely held assumption underpinned one of the most common concerns expressed during focus groups and interviews, namely that a release would lead to significant increases in the *Ae. aegypti* population, with residents being overrun with dengue or bacterially-infected mosquitoes and, consequently, exposed to much greater levels of biting and possible infection [37].

Importantly, sharing the results of studies allowed us to explain to residents that *Ae. aegytpi* cannot fly far and that they prefer to lay their eggs on the sides of containers, such as those we provided examples of. This helped to challenge ideas about the ubiquity of these insects in the landscape. It also made it clear to people that if they supported a release, the mosquitoes would be in and around their homes, which helped to ensure that they were better informed about what was being asked of them.

These findings indicated that knowledge of the disease and its transmission was very poor, explained in part by the mobility of the local population. Lay knowledge included very particular understandings and assumptions about the disease and the mosquito that were unique to the disease history of the region [37]. Clearly, if residents were to understand and fully assess the acceptability of the *Wolbachia* strategy and the field trials, specific information addressing these issues and local understandings would need to be provided alongside details about the project, the bacterium, its capacity to invade insect populations and its safety.

### The history of biological control in the region and its implications

Although a description of the process used to introduce the bacterium into the mosquito was given during all interviews and focus groups, the *Wolbachia* strategy was rarely associated with genetic modification. Instead, participants (without any prompting from research staff) frequently compared it to biological control programs, most commonly the introduction of the cane toad (*Bufo marinus*) and the use of the *myxoma* virus for rabbit control. The cane toad *Bufo marinus* was first released in Australia in the 1930s, near the township of Gordonvale, one of the potential release sites for the project. It is an infamous example of biological control gone wrong and in the 2009 telephone survey, when respondents were asked “Can you think of any examples of biological pest control” 62% answered “the cane toad”. In interviews and focus groups the cane toad was consistently held up as a cautionary tale, an example of the limitations of scientific knowledge and the unpredictable or unknowable effects of biological control and new technologies.

Furthermore, in reviewing the history of biological control in Australia and identifying which programs were likely to be familiar to local residents (Table S2), it became apparent that earlier programs had focused on pest management, involved the introduction of insects or invertebrates and had been implemented on farms or in forested areas [34]. This was significant because the *Wolbachia* method was intrinsically different in several important respects. The introduced biological control agent – the bacterium – was invisible to people, yet present in a well-known disease vector, *Ae. aegypti*, which had to be released in urban areas. This, coupled with the complexity of lay understandings of bacteria and negative perceptions of biological control, suggested that residents were likely to have concerns about the *Wolbachia* strategy's effectiveness, safety, its potential to be transferred to other species (particularly humans) or to cause some kind of unexpected harm. These results suggested that transference, safety and effectiveness would require a serious and detailed response and that differences in the current (as opposed to the past) regulatory environment would need to be emphasized.

### Controlling dengue in the region and the acceptability and non-acceptability of *Wolbachia*

The safety of the bacterium for people and the environment over the short and long term were the most serious and frequent concerns identified in the social research (see Table S4). In the 2009 telephone survey almost all respondents indicated that it was “important” to “very important” that it should not affect people, other insects or other animals (Table S4). Discussions in the focus groups and interviews tended to center on the potential for transference of the bacterium to people and the environment, broadly defined. The mechanisms of transference that participants explored included through feeding or biting behavior, and accidental ingestion or physical contact with the insect during any of its life stages, including its skin cells and feeding apparatus.

Concerns about transference of the bacterium into the environment, identified broadly as including soil, water and all organisms (especially native species) also dominated discussions. The potential mechanisms for transference included predation, biting or feeding behavior, exposure to the bacterium within and outside of the mosquito's body, and to the mosquito itself (alive or dead). This was further confirmed in the 2009 telephone survey where respondents were asked to rank the importance of a range of safeguards relating to the development and implementation of the *Wolbachia* strategy (Table S4). Almost all, 96%, stated that “it should not affect or be able to spread to other insects,” and 98% that “it should not affect or be able to spread to animals” (Table S4). There were no significant differences in these results by sex, length of residence or age.

Effectiveness of the *Wolbachia* strategy in the short and long term, the reliability of the research and, ultimately, trust in science, scientists and government were the next most common and most significant concerns identified across the qualitative research. In the 2009 survey, 78% said that *Wolbachia* should not be able to spread outside of Australia and 54% felt that it was “important” or “very important” that the control method was humane (Table S4). Analysis of the interview and focus group data indicated a desire to see the scientific results assessed independently and the method evaluated and approved by the relevant government department or regulator. In response to these early findings, in the March 2010 telephone survey residents were asked “If the *Wolbachia* method also received approval from a government regulator, would you feel comfortable about its use in the Cairns area to control dengue fever?” Overall, 86% of people answered “Yes”, 7% “No”, and 7% “unsure/don't know”.

Although the scientific team was confident about the safety of the strategy and was able to use current research and literature to respond to these concerns, we decided that this might not be sufficient to reassure the community who, in some instances, expected to be provided with experimental data that would directly assess those risks. Several new experiments were undertaken, including examination of the potential for *Wolbachia* to be passed into the human bloodstream through the mosquito's saliva during feeding, and the testing of *Wolbachia's* capacity to be transferred from mosquitoes to predator and non-predator species common to the local Cairns environment [36], [37], [38].

### Community expectations and requirements for engagement and authorization

A number of recurring and prominent issues emerged around expectations for engagement and authorization. When discussing community engagement in the focus groups and in-depth interviews, participants invariably noted the lack of public awareness and knowledge about dengue, the complexity of the *Wolbachia* method and its differences from current control measures. They consistently stated that every effort should be made to ensure residents were informed about the disease, current control measures and the research, and to engage as many people as possible, well before a release. Results from the 2009 telephone survey also indicated strong support for the provision of information on the science behind the program (86%) and for public consultation about new biological control programs (82%) (see Table S5). More than half of those surveyed supported public involvement in decision making processes relating to the *Wolbachia* program (Table S5). There were no significant differences in terms of age, length of residence, history of dengue or education, although more males believed public involvement in decision making was “not at all important”.

Residents shared ideas and expectations about engagement, the forms it should take, the priorities it should address, and what would constitute authorization. The most popular mechanism was face-to-face presentations, like those used in the focus groups, which also provided time for those present to ask questions of the scientists, reflect on their answers and hear other community members' views. Residents expected multiple points of contact with the program, including community presentations, web site, newsletter, and general media coverage that would keep them informed, update them on new results and allow time for people to digest and consider their response. Identifying their needs and expectations and exploring ways of incorporating them into the engagement strategy was important in establishing trust and showing respect.

Participants identified that working directly with local residents, leaders and civic groups that were respected and trusted was the most appropriate to way to engage and build awareness in this context. Another common theme was the issue of trust and the importance of being honest, open, respectful and transparent in all communication because local residents can, as one participant expressed it, “… be suspicious of engagement that looks too polished or appears to be trying to sell people something” (Focus Group 1; 2008). Residents were well aware of the limitations of knowledge, including scientific knowledge and expected to hear where these gaps were and what their implications would mean.

Along with the size and diversity of the Cairns population, these results suggested that a large, broad-based engagement strategy would be needed, given (a) the limited knowledge of dengue fever, (b) the relative newness (in the public consciousness) of using biological control in public health, (c) strong indications that residents would expect to be engaged early, honestly and extensively, (d) the need to communicate the “*Wolbachia* story” effectively to a largely non-scientific audience, and (e) limited knowledge of successful biological control. It was likely that the project would be better received and understood locally through face-to-face presentations delivered in the community and open invitation community meetings. This would also allow project staff to connect with residents, build relationships, hear their questions and address their concerns. Given the mobility of the population, the strategy would need to reach a broad cross-section of Cairns residents and a significant number of people from Gordonvale and Yorkeys Knob through additional media support and attendance at local events.

In May 2009 a public engagement strategy was developed, including communication materials to meet these and other parameters, and with implementation from June 2009 to June 2010. The strategy included a series of new scientific experiments, an independent assessment of the science, the establishment of a reference group, and increased and extensive engagement with the wider community. The forms of engagement used, excluding media, are detailed below (Table S6). Media coverage was not extensive; on average a story appeared in the local or national press once every 10 weeks during this period.

We also surveyed residents' perceptions of the acceptability of a range of control measures, including *Wolbachia*. In the March 2009 telephone survey, 77% identified *Wolbachia* as the most acceptable means of dengue control, followed by insecticide use (67%) (Table S7). Those aged 55–64 considered the use of an insect bacterium more unacceptable but there were no other significant differences in terms of gender, education or length of residence. By March 2010 support for *Wolbachia* had increased to 85%, with insecticide use at 66% (Table S8). In the 2009 survey, 2% of respondents found *Wolbachia* “very unacceptable” and 9–11% “unacceptable” (Table S7), but by the 2010 survey this had decreased to 0% and 7–8% respectively (Table S8). In 2010, acceptability for the use of an insect bacterium to prevent transmission was lower among those 18–24 years but there were no other significant differences in terms of gender, education or length of residence.

During the engagement phase (2009–2010) an anonymous questionnaire was distributed at the end of each presentation to track responses to dengue control and the project and evaluate any new developments (e.g. the completion of an independent risk assessment in late 2009). As of June 2010, 84% of respondents indicated they would support the use of *Wolbachia-*infected *Ae. aegypti* if (a) it has regulatory oversight, (b) they are engaged, informed and updated about the progress of the science and the release, and (c) it is shown to be safe for people and the environment by the independent risk assessment carried out by the CSIRO (Australia's Commonwealth Scientific and Industrial Research Organization) [39].

## Discussion

Public involvement in biological control initiatives in Australia has been increasing in recent decades, as have debates about the nature and scale of this involvement [40], [41], [42], [43], [44], [45], [46]. However, engagement is often weakest at the development and consultation phase and strongest around the intervention stage, through activities such as education campaigns or bio-agent distribution [47]. Participation in attitudinal surveys has also become more common and in some instances this has had an impact on policy decisions around the use of particular bio-agents. Significantly, activity appears to be strongest in the emergence of initiatives that are often highly localized, especially those involving the release of an agent or the removal of pest species, such as *Tilapia* spp.

Scientists and funding bodies involved in the current and similar programs recognize that early engagement [4], [6], community enablement and authorization are essential to these programs [7], [8], [9], [10], [11]. Some have posited that “there is no explicit body of community engagement knowledge to which researchers can turn for guidance about approaches that are most likely to be effective in different contexts, and why” [48]. Research from a similar program in Mexico has developed an early framework that explores issues around site selection, regulatory approvals and developing administrative frameworks for decision making alongside public consultation [6].

As noted earlier, the approach described here is underwritten by a number of key insights drawn from the extant anthropological literature. First, many health interventions fail because of a limited understanding of lay knowledge of disease and the broader sociopolitical context. Second, the public will have *particular* concerns and questions about the science and expectations about engagement and authorization that will vary from place to place and which need to be identified and addressed. Third, using longer-term systematic social research provides a *more* reliable way of exploring these issues, enabling participation and the development of engagement strategies and communication materials tailored to the needs, knowledge and expectations of diverse and complex communities at potential release sites. Fourth, those citizens most affected by the use or trial of new disease control methods should be engaged early and given access to culturally appropriate, understandable and accessible information from which they can decide how they want the disease to be managed and whether to support a new initiative.

As the results presented above show, the approach developed here has many advantages. It involves community members at all stages (research and engagement) but does not have the limitations of time and lack of sensitivity to context often associated with Participatory Action Research, Community-Based Participatory Research or surveys. It also provides insight into the history, composition and sociopolitical complexity of the multiple publics at a potential release site, which is invaluable in determining the scale and nature of future engagement strategies and is not available through any other mechanism. It can highlight features of lay understandings of disease, disease etiology, ethno-entomology and past biological control that may impact on public perceptions of the disease or the project, and provide insights into how these might be challenged or addressed. Cairns residents have rarely been asked for this kind of input, so our research activities and the implementation of the broad based engagement strategy (see Table S6) over 12 months was critical to building relationships and developing trust. It also showed a commitment to the forms and level of engagement people had expressly asked for. The establishment of a reference group in 2010 was the key to the formal engagement process and to providing formal collaboration and a central role in decision making for local residents.

As noted above, some residents requested an independent review of the science and some form of external oversight of the project. By knowing this well in advance of the formal engagement and a release, we were able to provide time and space to arrange for an independent risk assessment to be completed. Recurring questions or concerns were fed back to the scientific team, who undertook new experiments and prepared responses to questions during community presentations that were adapted for use in communication materials and on the web site.

A key lesson from the project is that longer-term research using mixed methods is essential to identifying concerns about a program, and this in turn provides opportunities to develop precise, considered, educationally and culturally appropriate responses and resources. This helped us to create a consistent message, address concerns and lay understandings sensitively, confidently and effectively and build trust and show respect, because residents' concerns were being taken seriously and not simply dismissed based on current knowledge. Of course, the approach described here may not be feasible for all programs, especially those limited by time or budget constraints (we implemented it over 2 years with 1.5 full-time staff members). It does, however, provide possibilities for those where the science is at a similar stage of development and there is a commitment to early public involvement, enablement and engagement. It has been used successfully in two countries.

### Summary

In sum, this approach used long-term research to facilitate the development of an engagement strategy and communication materials tailored to the specific needs of the communities at the release sites. It is distinguished by its focus on sensitivity to the local sociopolitical context, to lay knowledge of disease and past control initiatives and to uncovering expectations and concerns regarding the strategy, engagement and authorization. Because it was based on long-term, systematic social research that involved the public at every step, it is likely to be a more reliable mechanism for communicating the nature of the project and what is being asked of the community, and for developing trust and authorization for a release,

This study indicates that efforts to embrace the call for more ethical public participation and engagement in science need to develop engagement strategies and communication materials that are tailored for and comprehensible to the multiple publics at a given field site. If projects plan to use open field trials, they need to be aware of and responsive to the needs, expectations, concerns, desires and knowledge of the communities whose backyards they hope to use as open laboratories. Using long-term social research methods and attempting to understand lay knowledge, rather than dismissing it as non-scientific or wrong can greatly aid the transmission of knowledge about new scientific endeavors, such that residents are enabled to participate, critique, assess and determine whether they want these strategies to be trialed or implemented in their backyards and communities.

Other approaches, such as not undertaking social research, relying on quick, short-term techniques, using a “one size fits all” approach to participation or engagement, or choosing to “sell” these programs to communities through media campaigns, risk undermining the broader political aims of community participation and engagement and the goodwill that the public bring to these encounters. In Cairns, these approaches would be met with suspicion and, as such, would struggle to build support over the long term. Residents know there are limitations to scientific knowledge and they do not see the *Wolbachia* strategy as just another biological control intervention. They expect to be fully informed and engaged about the science, the project and any future releases and they also want the opportunity to ask questions, engage in critique, determine how dengue is managed and say no. While the language local residents use to express their questions and concerns may be different to that of the scientists, it is important to note that their issues mirrored many of the research questions being asked by scientists both within and outside of the project.

## Supporting Information

Table S1 **Potential release sites in Australia.**
(DOC)

Table S2 **Issues, Methods and Recruitment.**
(DOC)

Table S3 **Excerpt from Social Demographic Data.**
(DOC)

Table S4 **Importance of safeguards associated with** ***Wolbachia*** **-infected** ***Aedes aegypti*** **(%).**
(DOC)

Table S5 **Public expectations regarding engagement and participation (%).**
(DOC)

Table S6 **Forms of Engagement - June 2009 to June 2010.**
(DOC)

Table S7 **Safety and acceptability of control methods (%).**
(DOC)

Table S8 **Safety and acceptability of control methods (%).**
(DOC)
