# Supplementary material for: The Importance of Long-Term Social Research in Enabling Participation and Developing Engagement Strategies for New Dengue Control Technologies
Source: PLoS Negl Trop Dis. 2012 Aug 28;6(8):e1785. doi: 10.1371/journal.pntd.0001785 (PMC3429396; doi:10.1371/journal.pntd.0001785)
Supplement: Table S1 — Potential release sites in Australia. (DOC) [file pntd.0001785.s001.doc]

Table 1: Potential release sites in Australia

| **Potential release sites – Cairns, Australia** | | | | |
| --- | --- | --- | --- | --- |
| Suburb | History | Population | Location | Median Household Income (Australia $1,171) |
| GORDONVALE | Located on the traditional homelands of Yidinyi People. Established as a sugar-producing town in 1896 and continues to produce and process sugar cane. It emerged as a small township in the early 20^th^ century, and while today it is a suburb of Cairns, it has a strong local identity and links to local agricultural and resource industries | 4,420 | 23km south of Cairns city | $1,005 |
| YORKEYS KNOB | Located on the Traditional homelands of the Yirrganydji people, Yorkeys Knob emerged in the mid 20^th^ century as a small, residential beachside suburb whose population has grown considerably from 200 people in 1958. It is one of the few beach areas to have resisted high levels of tourism development. | 2,684 | 22km north of Cairns city | $897 |
