# Supplementary material for: The Importance of Long-Term Social Research in Enabling Participation and Developing Engagement Strategies for New Dengue Control Technologies
Source: PLoS Negl Trop Dis. 2012 Aug 28;6(8):e1785. doi: 10.1371/journal.pntd.0001785 (PMC3429396; doi:10.1371/journal.pntd.0001785)
Supplement: Table S2 — Issues, Methods and Recruitment. (DOC) [file pntd.0001785.s002.doc]

Table 2: Issues, Methods and Recruitment

| **Initiative** | **Method** | **Target Population** | **Recruitment & Sampling** | **Analysis** |
| --- | --- | --- | --- | --- |
| ***Community profile*** | Historical research | Cairns Region | None |  |
|  | Analysis of Australian Census data | City, Region & National figures | None |  |
|  | In depth interviews (A) (n=10) | Local leaders | *Purposive* Invited | Field notes NVivo |
| ***History of disease, management and bio control*** | Review local & national literature on dengue fever and mosquito control | None | None |  |
|  | Review national & international. literature on biological control | None | None |  |
|  | Focus groups (A) n=2, with 19 participants | Mosquito control & health staff | *Purposive* Invited | Recorded NVivo |
|  | Informal ethnographic interviews (n=40) | Local residents | *Convenience* | Field notes NVivo |
| ***Stakeholder contact list*** | In-depth interviews (A) (n=10) | Local leaders | *Purposive* Invited | Field notes NVivo |
|  | Focus groups (A) n=2, with 19 participants | Mosquito control & health staff | *Purposive* Invited | Recorded NVivo |
| ***Lay knowledge of dengue*** | Informal ethnographic interviews (40) | Local residents | *Convenience* | Field notes NVivo |
|  | In-depth interviews (B) (40) | Local residents Notices in local newspaper | *Stratified* by age and gender | Field notes NVivo |
|  | Focus groups (B) n=9, 82 participants | Local residents Notices in newspaper | *Stratified* by age and gender | NVivo |
|  | Telephone survey 2009 (n=300) | Randomly generated phone numbers | *Randomized* *representative* sample | NVivo SPSS |
|  | Health Authority survey data from 2004, 2007, 2008 (n=1200) | Randomly generated phone numbers | *Randomized* *representative* sample | NVivo SPSS |
| ***Acceptability, and safety, engagement and authorization*** | In-depth interviews (A) (n=10) | Local leaders | *Purposive* Invited | Field notes |
|  | Focus groups (B) n=9, 82 participants | Local residents Notices in local newspaper | *Stratified* by age and gender | NVivo |
|  | Telephone survey 2009 (n=300) | Randomly generated phone numbers | *Randomized* *representative* sample | NVivo SPSS |
|  | Telephone survey 2010 (n=300) | Randomly generated phone numbers | *Randomized* *representative* sample | NVivo SPSS |
