# Supplementary material for: The Importance of Long-Term Social Research in Enabling Participation and Developing Engagement Strategies for New Dengue Control Technologies
Source: PLoS Negl Trop Dis. 2012 Aug 28;6(8):e1785. doi: 10.1371/journal.pntd.0001785 (PMC3429396; doi:10.1371/journal.pntd.0001785)
Supplement: Table S3 — Excerpt from Social Demographic Data. (DOC) [file pntd.0001785.s003.doc]

Table 3: Excerpt from Social Demographic Data

| **Social Demographic Data – Australian National Census 2006** | | | | | |
| --- | --- | --- | --- | --- | --- |
|  | Indigenous residents | Over 65years |  | One-parent families | Families with no children |
| Gordonvale | 11.7% | 12.4% | Yorkeys Knob | 21.7% | 46.7% |
| Yorkeys Knob | 5.2% | 8.7% | Nationally | 15.8% | 37.2% |
