# Supplementary material for: The Importance of Long-Term Social Research in Enabling Participation and Developing Engagement Strategies for New Dengue Control Technologies
Source: PLoS Negl Trop Dis. 2012 Aug 28;6(8):e1785. doi: 10.1371/journal.pntd.0001785 (PMC3429396; doi:10.1371/journal.pntd.0001785)
Supplement: Table S4 — Importance of safeguards associated with Wolbachia -infected Aedes aegypti (%). (DOC) [file pntd.0001785.s004.doc]

Table 4: Importance of safeguards associated with *Wolbachia*-infected *Aedes aegypti* (%)

| **2009 telephone survey (n=300)** |  |  |  |  |  |  |
| --- | --- | --- | --- | --- | --- | --- |
| **If there was a way to use this insect bacteria to control the dengue mosquito, how important would the following safeguards be in developing and implementing such a program?** | **Very important** | **Important** | **Not that important** | **Not at all important** | **Don’t know/Not sure** | **TOTAL** |
| The biological agent (insect bacteria) should not affect people | 86 | 13 | 0 | 0 | 1 | 100% |
| The biological agent (insect bacteria) should not affect or be able to spread to other insects | 75 | 21 | 1 | 1 | 2 | 100% |
| The biological agent (insect bacteria) should not affect or be able to spread to animals | 81 | 17 | 1 | 1 | 0 | 100% |
| The biological agent (insect bacteria) should not be able to spread outside of Australia | 50 | 28 | 16 | 1 | 5 | 100% |
| The biological control method should be humane | 27 | 27 | 14 | 29 | 3 | 100% |
