# Supplementary material for: The Importance of Long-Term Social Research in Enabling Participation and Developing Engagement Strategies for New Dengue Control Technologies
Source: PLoS Negl Trop Dis. 2012 Aug 28;6(8):e1785. doi: 10.1371/journal.pntd.0001785 (PMC3429396; doi:10.1371/journal.pntd.0001785)
Supplement: Table S5 — Public expectations regarding engagement and participation (%). (DOC) [file pntd.0001785.s005.doc]

Table 5: Public expectations regarding engagement and participation (%)

| **2009 telephone survey (n=300)** |  |  |  |  |  |  |  |
| --- | --- | --- | --- | --- | --- | --- | --- |
| **If there was a way to use this insect bacteria to control the dengue mosquito, how important would the following safeguards be in developing and implementing such a program?** | **Very important** | **Important** | **Not that important** | **Not at all important** | **Don’t know/ Not sure** | **TOTAL** | **POSITIVE** |
| The public should be provided with information on the science behind the biological control program | 40 | 46 | 10 | 2 | 2 | 100% | 86 |
| The public should be consulted about new biological control programs | 38 | 44 | 14 | 1 | 3 | 100% | 82 |
| The public should be involved in decision making processes relating to such a biological control program | 22 | 34 | 29 | 10 | 5 | 100% | 56 |
