# Supplementary material for: The Importance of Long-Term Social Research in Enabling Participation and Developing Engagement Strategies for New Dengue Control Technologies
Source: PLoS Negl Trop Dis. 2012 Aug 28;6(8):e1785. doi: 10.1371/journal.pntd.0001785 (PMC3429396; doi:10.1371/journal.pntd.0001785)
Supplement: Table S6 — Forms of Engagement - June 2009 to June 2010. (DOC) [file pntd.0001785.s006.doc]

Table 6: Forms of Engagement – June 2009 to June 2010

| **Forms of engagement – June 2009 to June 2010** | |
| --- | --- |
| Formal presentations (30–60 minutes) | 115 |
| Number of participants (residents from environmental, health, education, local government, Indigenous, migrant, sporting and civic groups) | 1500 |
| Informal presentations/meetings (to local leaders and key organizations) | 134 |
| Number signed up for and receiving the CE Newsletter every 3 months | 1200 |
| Invitations from community to attend events | 15 |
| Regular stall at two local monthly markets | 8 |
| Presentations and tours of the Mosquito Research Facility | 20 |
| Initiated development of an upper primary level (ages 10–12 years) dengue fever education subject that was adopted by the Department of Education (Queensland) and is now being taught in a number of schools in the region |  |
