# Supplementary material for: The Importance of Long-Term Social Research in Enabling Participation and Developing Engagement Strategies for New Dengue Control Technologies
Source: PLoS Negl Trop Dis. 2012 Aug 28;6(8):e1785. doi: 10.1371/journal.pntd.0001785 (PMC3429396; doi:10.1371/journal.pntd.0001785)
Supplement: Table S7 — Safety and acceptability of control methods (%). (DOC) [file pntd.0001785.s007.doc]

**Table 7: Safety and acceptability of control methods (%)**

| **2009 telephone survey (n=300)** |  |  |  |  |  |  |  |
| --- | --- | --- | --- | --- | --- | --- | --- |
| **Can you please tell me how safe and acceptable you think the following methods are for controlling the dengue mosquito?** | **Very acceptable** | **Acceptable** | **Unacceptable** | **Very unacceptable** | **Don’t know/ Not sure** | **TOTAL** | **POSITIVE** |
| 1. Spraying insecticide inside your home that kills the dengue mosquito and other insects | 7 | 60 | 23 | 7 | 3 | 100% | 67 |
| 2. Spraying insecticide around your garden that kills the dengue mosquito and other insects | 8 | 57 | 27 | 3 | 5 | 100% | 65 |
| 3. Introducing an insect bacteria into the mosquito which will prevent it from transmitting dengue to people | 23 | 54 | 9 | 2 | 12 | 100% | 77 |
| 4. Using an insect bacteria to shorten the lifespan of the mosquito which will prevent it from transmitting dengue to people | 23 | 55 | 11 | 2 | 9 | 100% | 78 |
| 5. Introducing a parasite to mosquito breeding containers that will kill the mosquito larvae | 14 | 42 | 26 | 7 | 11 | 100% | 56 |
| 6. Releasing genetically modified mosquitoes that cannot transmit dengue to people | 6 | 29 | 33 | 19 | 13 | 100% | 35 |
