# Supplementary material for: The Importance of Long-Term Social Research in Enabling Participation and Developing Engagement Strategies for New Dengue Control Technologies
Source: PLoS Negl Trop Dis. 2012 Aug 28;6(8):e1785. doi: 10.1371/journal.pntd.0001785 (PMC3429396; doi:10.1371/journal.pntd.0001785)
Supplement: Table S8 — Safety and acceptability of control methods (%). (DOC) [file pntd.0001785.s008.doc]

Table 8: Safety and acceptability of control methods (%)

| **2010 telephone survey (n=300)** |  |  |  |  |  |  |  |
| --- | --- | --- | --- | --- | --- | --- | --- |
| **Can you please tell me how safe and acceptable you think the following methods are for controlling the dengue mosquito?** | **Very acceptable** | **Acceptable** | **Unacceptable** | **Very unacceptable** | **Don’t know /not sure** | **TOTAL** | **POSITIVE** |
| 1. Spraying insecticide inside your home that kills the dengue mosquito and other insects | 11 | 54 | 32 | 1 | 2 | 100% | 65 |
| 2. Spraying insecticide around your garden that kills the dengue mosquito and other insects | 11 | 55 | 31 | 1 | 3 | 100% | 66 |
| 3. Introducing an insect bacteria into the mosquito which will prevent it from transmitting dengue to people | 32 | 52 | 7 | 0 | 8 | 100% | 84 |
| 4. Using an insect bacteria to shorten the lifespan of the mosquito which will prevent it from transmitting dengue to people | 33 | 52 | 8 | 0 | 8 | 100% | 85 |
| 5. Introducing a parasite to mosquito breeding containers that will kill the mosquito larvae | 19 | 47 | 20 | 1 | 13 | 100% | 66 |
| 6. Releasing genetically modified mosquitoes that cannot transmit dengue to people | 13 | 37 | 33 | 3 | 14 | 100% | 50 |
